# Assessment of Paclitaxel Induced Sensory Polyneuropathy with “Catwalk” Automated Gait Analysis in Mice

**DOI:** 10.1371/journal.pone.0076772

**Published:** 2013-10-15

**Authors:** Petra Huehnchen, Wolfgang Boehmerle, Matthias Endres

**Affiliations:** 1 Klinik und Hochschulambulanz für Neurologie, Charité Universitätsmedizin Berlin, Berlin, Germany; 2 Cluster of Excellence NeuroCure, Charité Universitätsmedizin Berlin, Berlin, Germany; 3 Center for Stroke Research Berlin, Charité Universitätsmedizin Berlin, Berlin, Germany; Imperial College London, Chelsea & Westminster Hospital, United Kingdom

## Abstract

Neuropathic pain as a symptom of sensory nerve damage is a frequent side effect of chemotherapy. The most common behavioral observation in animal models of chemotherapy induced polyneuropathy is the development of mechanical allodynia, which is quantified with von Frey filaments. The data from one study, however, cannot be easily compared with other studies owing to influences of environmental factors, inter-rater variability and differences in test paradigms. To overcome these limitations, automated quantitative gait analysis was proposed as an alternative, but its usefulness for assessing animals suffering from polyneuropathy has remained unclear. In the present study, we used a novel mouse model of paclitaxel induced polyneuropathy to compare results from electrophysiology and the von Frey method to gait alterations measured with the Catwalk test. To mimic recently improved clinical treatment strategies of gynecological malignancies, we established a mouse model of dose-dense paclitaxel therapy on the common C57Bl/6 background. In this model paclitaxel treated animals developed mechanical allodynia as well as reduced caudal sensory nerve action potential amplitudes indicative of a sensory polyneuropathy. Gait analysis with the Catwalk method detected distinct alterations of gait parameters in animals suffering from sensory neuropathy, revealing a minimized contact of the hind paws with the floor. Treatment of mechanical allodynia with gabapentin improved altered dynamic gait parameters. This study establishes a novel mouse model for investigating the side effects of dose-dense paclitaxel therapy and underlines the usefulness of automated gait analysis as an additional easy-to-use objective test for evaluating painful sensory polyneuropathy.

## Introduction

Many drugs used in clinical tumor chemotherapy induce peripheral neuropathy (PNP), which not only increases the burden of disease, but sometimes is also dose limiting and thus detrimental to therapy [1]. In patients, toxic injury of peripheral nerves typically manifests as a predominantly axonal neuropathy, with often painful symptoms distributed in a length-dependent “glove and stocking” fashion. The underlying pathomechanisms are still poorly understood and treatment options are limited, warranting further research. Various animal models of chemotherapy induced polyneuropathy (CIPN) have therefore been developed and characterized (reviewed by [2]).

The most common finding in these models of painful neuropathies is mechanical allodynia, a symptom of an underlying sensory PNP [3]. In animals mechanical allodynia can be assessed by determining the response threshold to a defined non-painful mechanical stimulus. Technically this is usually measured with a series of increasingly stiff von Frey filaments [4] or an electronic pressure-meter [5]. In many animal studies the development of mechanical allodynia, as signified by a reduced mechanical withdrawal threshold, has been used to quantitate and describe the course of sensory neuropathy. One caveat of detecting mechanical allodynia with the von Frey hair method is that the definition of a response may vary between investigators and many different ways of performing this test have been published [4], [6], [7]. This leads to difficulties in the interpretation of results from different groups and even between investigators within one laboratory.

To overcome these limitations automated gait analysis with the Catwalk method has been proposed as a more objective tool for evaluating sensory mononeuropathy [8]. In this test, animals walk freely through a glass bottom tunnel, where placement of a paw on the glass elicits an intensity-dependent signal. Gait patterns are recorded with a camera and subjected to automated software-based analysis. Rats suffering from chronic constriction injury of the sciatic nerve demonstrated alterations of gait parameters that correlated closely with mechanical withdrawal thresholds obtained by the von Frey method [8]. The Catwalk test has since been used successfully to evaluate animals with unilateral nerve lesions [9], [10] as well as inflammatory pain states [11], [12].

We hypothesized that the Catwalk method might also be useful to quantify sensory PNP in a CIPN animal model. Paclitaxel is a frequently used cytostatic agent which often causes a painful sensory PNP in patients [13]. Recently, dose-dense paclitaxel therapy with more frequent application of a reduced standard dose has proved beneficial in gynecological tumors such as ovarian and breast cancer [14], [15]. In the present study, we thus developed a novel mouse model which mimics key aspects of dose-dense paclitaxel therapy. We characterized the phenotype of these animals with established methods like the von Frey and electrophysiological tests and compared results to gait alterations measured with the Catwalk apparatus. In an additional set of experiments, we evaluated the effects of gabapentin treatment on gait parameters as well as mechanical allodynia.

## Materials and Methods

### Ethics Statement

This study conformed to government and institutional animal welfare guidelines and was approved by the official animal ethics committee of Berlin (Landesamt fuer Gesundheit und Soziales, Berlin, Germany) prior to the execution of the experiments. The protocol was optimized in accordance with the three R principles and every effort was made to minimize suffering.

### Animals

A total of 85 nine-week old male C57BL/6J mice from Charles River (Sulzfeld, Germany) were used for this study. After delivery, animals were assigned to cages using randomly generated numbers in order to reduce possible litter effects. Cages were likewise allocated to treatment groups with randomly generated numbers. Mice were housed in groups of five in an enriched environment and allowed food and water ad libitum. The animals were maintained on a 12∶12 hour light/dark cycle (7 am-7 pm) and behavioral testing was conducted between 10 am and 6 pm. If an injection was administered on the same day as behavior tests, it was administered after all testing had been completed, with the exception of gabapentin which was applied 1 hour prior to behavioral testing [16]. Daily the general wellbeing of the mice was assessed and their weight recorded.

### Drug Injection protocol, group sizes

**Figure 1:**
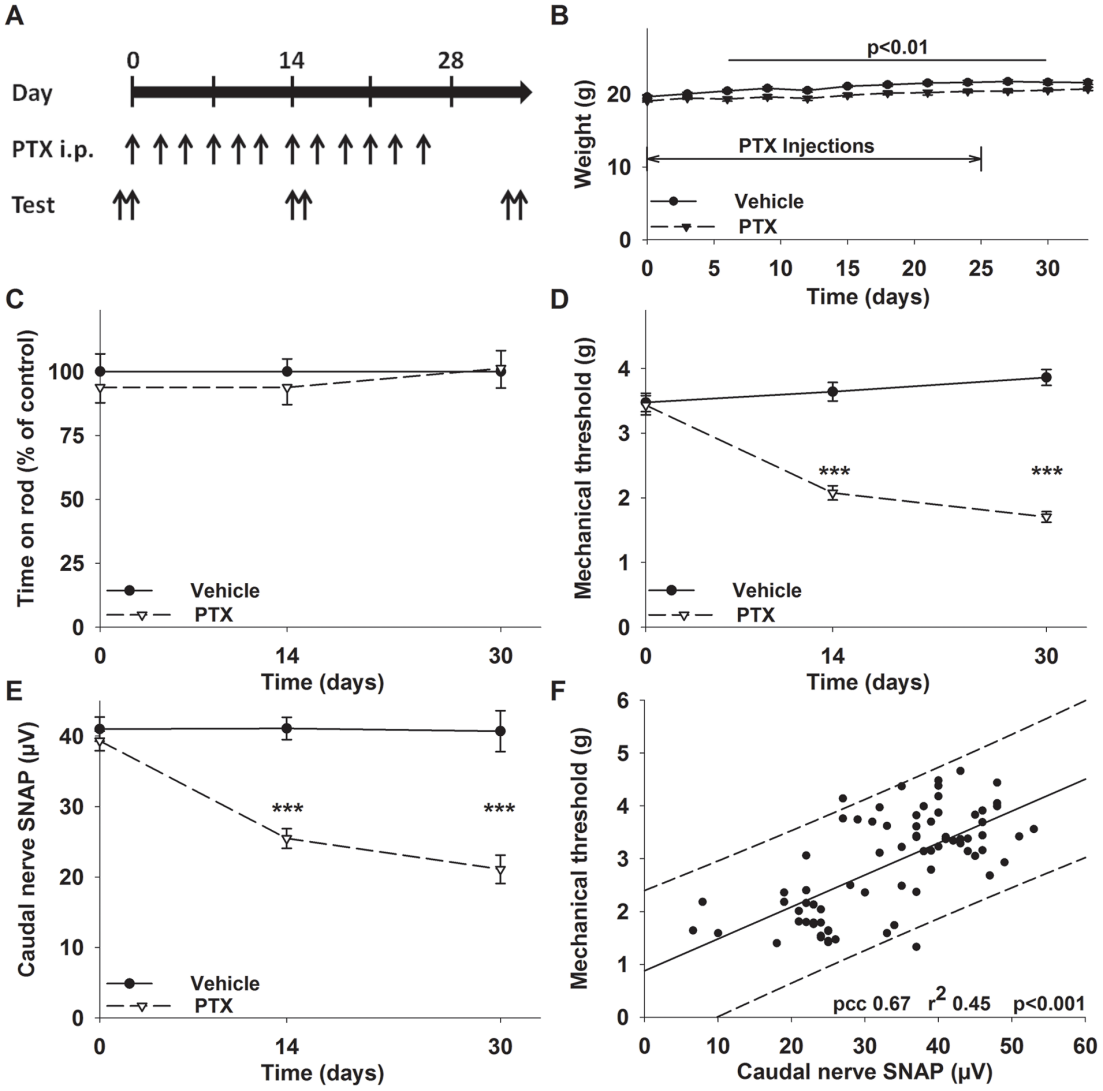
Dose-dense paclitaxel treatment induces a sensory polyneuropathy in mice. (A) Schedule of paclitaxel treatment and behavioral as well as electrophysiological testing. Animals received paclitaxel at a dose of 20 mg paclitaxel/kg BW three times per week over four weeks with a cumulative dose of 240 mg/kg BW. (B) Paclitaxel treated animals gained less weight than controls, but their weight quickly normalized after the last injection. (C) Motor coordination in the rotarod test was comparable in paclitaxel and vehicle injected animals. (D) Paclitaxel treated animals developed mechanical allodynia with a significantly reduced mechanical withdrawal threshold of the hind paws as well as a (E) predominantly axonal neuropathy with a diminished sensory nerve action potential amplitude (SNAP) of the caudal nerve. (F) Alterations of the caudal nerve SNAP showed a strong positive correlation with the mechanical withdrawal threshold (dashed line depicts the 95% prediction interval). *** p<0.001 compared to the control group at the same time point (One-way ANOVA).

Two different sets of experiments were performed: In the initial trial, we assigned 15 animals to the treatment and 10 animals to the control group, as previous studies reported a mortality of 25 to 50% for 20 mg/kg body weight (BW) paclitaxel i.p. in mice [17]. In the second experiment, animals were treated with gabapentin after induction of neuropathy with paclitaxel. We determined the group size of the second experiment based on the observations of the first experiment, with a desired power of 0.8 and an alpha level of 0.05 using SigmaPlot software (SigmaPlot, Systat, Richmond, CA). The calculated sample size with four different groups (vehicle/vehicle, vehicle/gabapentin, paclitaxel/vehicle, paclitaxel/gabapentin) was 15 animals per group. Paclitaxel (PTX, Sigma-Aldrich, Taufkirchen, Germany) was dissolved in Cremophor EL: Ethanol (1∶1) with a concentration of 6 mg/ml and diluted in sterile 0.9% NaCl solution to a final concentration of 2 mg/ml before intraperitoneal (i.p.) injection. Per treatment a dose of 20 mg PTX/kg BW in a total volume of 10 ml/kg BW was administered. Animals in the control group received an injection of vehicle (Cremophor EL: Ethanol, 1∶1, diluted in sterile 0.9% NaCl solution to a final concentration of 33.3% Cremophor EL: Ethanol). Mice received three injections per week, every other day, followed by a two day rest before the start of the next cycle. Over four weeks the animals thus received a total of 12 injections (Figure 1A). Gabapentin (Toronto Research Chemicals, Toronto, Ontario) was dissolved in sterile 0.9% NaCl solution to a final concentration of 10 mg/ml. Animals were treated with an i.p. injection of 100 mg/kg BW gabapentin (GBP) or 0.9% NaCl (vehicle) with an injection volume of 10 ml/kg BW daily. Behavior tests before the start of the intervention on day 26 and 27 as well as after seven and eight consecutive days of gabapentin treatment were compared.

### Behavior analysis

Behavior analysis was conducted on two consecutive days: On the first day, mice were tested with the Catwalk apparatus, allowed to rest and then (in the first experiment) tested on the rotarod. On the second day, the mechanical withdrawal threshold was measured with the von Frey method, followed by electrophysiological tests in anesthesia. Cages and animals were randomly selected for testing by a blinded experimenter. All behavior tests were carried out in a dedicated laboratory with soundproof chambers.

#### Rotarod

Motor coordination was assessed using the rotarod performance test. Mice were placed on a rotating rod in individual compartments, with walls on both sides and in front of them (TSE Systems GmbH, Bad Homburg, Germany). The speed of the rotating rod gradually increased starting at four rpm and reaching a maximum speed of 40 rpm in 300 seconds. Latency for the animal to fall off the rod was recorded by a floor sensor. Training was carried out for four days with three trials per day, with a daily increase in the maximum time spent on the rod from 70 s per trial on day one to 300 s per trial on day four, to allow mice to learn the task. During training, mice that fell off the rod within the designated time were gently placed back on the rod. Mice were brought back to their home cage from off the rod only, to prevent animals from exhibiting dropping behavior. The baseline was recorded on the last day of training by measuring the initial latency to fall off the rod. Post-injection testing was done at two time points (14 and 30 days after the first PTX injection) to determine if motor performance was affected. For each time point three trials were averaged.

#### Von Frey Hair test

Mechanical allodynia was measured with an electronic von Frey hair test as described previously [5]. The mice were placed under an inverted plastic cage with a wire-mesh floor. Investigators were trained to apply filaments to the center of the hind paw, gradually increasing pressure, for approximately five seconds. Poking either hind paw evoked a flexion reflex followed by a clear withdrawal response. The force applied to produce a withdrawal response was determined with a hand held force transducer fitted with a 0.5 mm^2^ polypropylene tip (IITC, Woodland Hills, CA). The mechanical withdrawal threshold in grams was automatically recorded when the paw was withdrawn. The maximum pressure applied was 10 g. Three time points were recorded in the first experiment (baseline, 14 and 30 days after the first PTX injection) and four in the second (baseline, 14 and 26 days after the first PTX injection as well as after eight consecutive days of gabapentin treatment), per time point five trials of each hind paw were averaged.

#### Catwalk

The Catwalk apparatus (Noldus Information Technology, Netherlands) consisted of a 1.3 m long black tunnel with a glass platform illuminated from within by total internal reflection of fluorescent light. If downward pressure was applied, the light was reflected and illuminated the stimulus. Underneath the glass a high-speed camera was mounted to record the walking patterns. The animals' home cages were placed at one end of the runway and mice were placed in the tunnel at the opposite end, thus walking freely across the runway into their home cage. The experiment was performed in a darkened room with red light and sheltered from noise. Analysis was performed using the Catwalk XT 8.1 Software. A trial was regarded as compliant by the software if the animal did not show a maximum speed variation greater than 60%. Six compliant trials were recorded for each animal and time point. A training period of four days was carried out prior to the first injection to acclimatize and train the animals in the task. Three time points were recorded in the first experiment (baseline: last day of training, 14 and 30 days after the first PTX injection) and four in the second (baseline: last day of training, 14 and 26 days after the first PTX injection as well as after seven consecutive days of gabapentin treatment). All trials marked by the software as compliant were reviewed manually: if the animal stopped or turned in mid-run the trials were not included in the analysis, thus a minimum of three to six validated runs were available per animal. As alterations induced by CIPN develop in a symmetrical fashion, we analyzed parameters for the left and right side together. To eliminate influences of walking speed on gait parameters [18], we adapted a previously published strategy [19] and used trials closest to the average walking speed of all animals in the experiment for further evaluation. This reduced the difference in walking speed between groups from up to 20% to below one percent.

### Electrophysiology

Nerve conduction velocity (NCV) and sensory nerve action potential amplitudes (SNAP) of the caudal nerve were recorded in isoflurane anesthesia (1.3% to 1.5% in O_2_) with a Dantec Keypoint electromyography system (Natus Medical Inc., Planegg, Germany). The protocol was adapted from Wang and colleagues [20]. In brief: stimulation electrodes were placed at the base of the tail with the recording electrodes five cm distal. A ground electrode was placed in between the stimulation and recording electrodes. 50 stimuli (0.1 ms) with supramaximal stimulation intensity and a frequency of 1Hz were averaged to measure SNAP and NCV at three time points (baseline: before injection, 14 and 30 days after the first PTX injection).

### Statistical analysis

Data is expressed as mean ± sem and the manuscript was written in accordance with ARRIVE guidelines [21]. Data analysis was completed before unblinding of the analyzer. Statistical analysis of the differences between treated versus control groups at multiple time points was performed by using a one- or two-way ANOVA with the Holm-Sidak post hoc test in case of normal distribution (normality test: Shapiro-Wilk; SigmaPlot, Systat, Richmond, CA). In the Catwalk analysis data points which deviated by more than two standard deviations from the sample mean were treated as outliers and the respective parameter pair (right/left) was excluded from analysis; this affected per experimental group between zero and (maximum) two animals. The number of outliers for each group and time point is summarized in Table S1. Only if statistical significance was detected between the treated and the control group, in otherwise untransformed data, was the respective parameter normalized to the control group and expressed as percent of baseline to obtain comparable results. Correlation analysis was performed by calculating the pearsons correlation coefficient (pcc) and significance using SigmaPlot. *p*<0.05 was considered statistically significant.

## Results

### Dose-dense paclitaxel treatment induces a sensory polyneuropathy in C57BL/6J mice

Given the increasing relevance of dose-dense paclitaxel therapy in the treatment of breast and ovarian cancer [14], [15], we aimed to establish a mouse model which mimics key aspects of this treatment. Typical human dosage is 12 applications of 80 mg paclitaxel per m^2^ body surface area [15], a dose which can be translated to approximately 25 mg/kg BW for mice [22]. To avoid serious hematological toxicity, we administered a slightly lower quantity of 20 mg/kg BW, a human equivalent dose of 65 mg/m^2^, which previously has been reported to be well tolerated in patients [23]. In analogy to clinical treatments, mice received a total of 12 injections with a cumulative dose of 240 mg/kg BW. We chose C57BL/6J mice due to their frequent use as genetic background in transgenic mice, even though this strain is only moderately susceptible to paclitaxel induced PNP [24].

Injections were administered every other day (three injections per week) with a two day rest before the next injection cycle (Figure 1A). This treatment was well tolerated, however mice receiving paclitaxel were slower to gain weight and during the treatment period weighed on average 1.1 g±0.1 g (F_(33,289)_ = 11.88; p<0.001; ANOVA) less than controls. The difference between the groups never exceeded 6%±1%, and after completion of paclitaxel treatment weights quickly recovered (Figure 1B).

Previously, alterations of motor coordination were observed with higher doses of paclitaxel [25], [26]. In this model of dose-dense therapy, performance of treated animals on the rotarod was comparable to the control group (Figure 1C). This is of interest, because despite of the high cumulative dose there was no detectable impairment of motor performance.

One hallmark of paclitaxel induced neuropathy in animals is mechanical allodynia. In line with previous reports [2], [27], paclitaxel treated animals developed a marked mechanical allodynia (F_(5,69)_ = 54.32; p<0.001; ANOVA) with a reduction of the mean mechanical withdrawal threshold, tested with an electronic von Frey device, by 38%±4% (p<0.001) after 14 days and 49% ±3% (p<0.001) compared to baseline after 30 days (Figure 1D).

Another common observation in animals with paclitaxel induced neuropathy is a decrease of the tail sensory nerve action potential [20], [28]. We observed a significant decrease (F_(5,69)_ = 25.78; p<0.001; ANOVA) of the caudal nerve SNAP by 35% ±5% (p<0.001) after 14 days and 46% ±5% (p<0.001) after 30 days compared to baseline (Figure 1E). Nerve conduction velocity showed a slight decrease (−9.5% and −7.4% after 14 respectively 30 days) which was not significant. This observation is comparable with results from patients who also develop electrophysiological alterations suggestive of a predominantly axonal PNP [13]. When caudal nerve SNAP was plotted against the sensory threshold, we observed a high degree of correlation (Pearson correlation coefficient (pcc) 0.67; r^2^ 0.45; p<0.001; Figure 1F). This lends support to the hypothesis that mechanical allodynia and electrophysiological alterations develop at the same time due to a common mechanism and are equally sensitive.

Taken together, we established an animal model of dose-dense paclitaxel therapy in male C57BL/6J mice which developed electrophysiological and behavioral alterations typical for a sensory PNP.

### Paclitaxel induced neuropathy can be detected with automated gait analysis

Automated gait analysis has been used previously to detect gait alterations induced by a mononeuropathy of the sciatic nerve [8], [9]. We hypothesized that this method could also be used to detect gait alterations in animals with PNP. One key disadvantage in the study of poly- versus mononeuropathy is the lack of an intraindividual control. We therefore compared paclitaxel treated animals with mice receiving vehicle injections. In the absence of an intraindividual control in either group, slight alterations of gait parameters between different time points were inevitable despite extensive training.

We next analyzed whether parameters that did not differ significantly between the two groups tended to change over time in the same direction, which would be suggestive for variability induced by environmental factors. As the alterations induced by paclitaxel typically occur in the lower limbs first and are distributed in a distal symmetrical fashion, we expected the hind paws to be more affected than the forepaws. We therefore chose 22 forepaw parameters (11 for right/left paw) which showed no statistical significance between the two groups at any time point and calculated the difference of group means for baseline versus early and early versus late time point respectively. Comparison of control versus paclitaxel treated animals for these parameters revealed a strong positive correlation with a pcc of 0.84 (r^2^ 0.71; p<0.001). This supports the hypothesis that slight variations observed between time points affect both treated and untreated animals alike. To describe the time course of different parameters we thus used control animals to normalize the data from treated animals.

**Figure 2:**
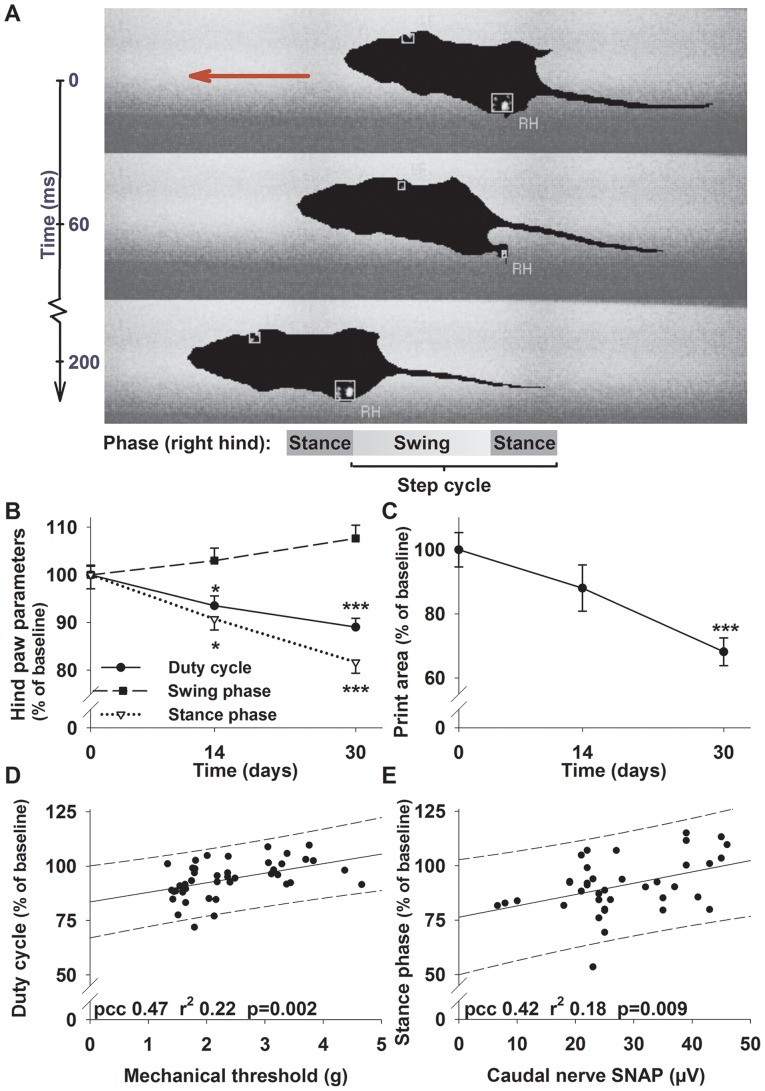
Paclitaxel induced polyneuropathy can be detected with the Catwalk method. (A) A typical Catwalk run; the animal crosses from right to left. Paws are automatically detected and labeled by the program, in this example right hind paw (RH) and the left forepaw. Examples of gait parameters for the right hind paw are displayed at the bottom of the graph in respect to the step cycle. (B) Animals treated with paclitaxel develop distinct gait alterations compared to vehicle controls. The swing phase (squares, long dashed line) increases, while the stance phase (open triangles, dotted line) and the duty cycle (circles, solid line) decreases. Duty cycle expresses the stance phase as a percentage of the entire step cycle (stand + swing). (C) The print area of the hind paws is also significantly reduced in neuropathic animals. (D) The duty cycle of the hind paws shows a small correlation with the mechanical withdrawal threshold of the animals. Similar results are obtained for the correlation of the (E) hind paw stance phase duration with the caudal nerve SNAP. Solid lines in (D-E) signify linear regression lines, while medium dashed lines depict the 95% prediction interval. * p<0.05; *** p<0.001; both compared to the control group at the same time point (One-way ANOVA).

We identified three parameters which were altered significantly in the hind paws of neuropathic animals: The swing phase was increased to 103% ±3% after 14 days and 107% ±3% of baseline after 30 days (both not significant; Figure 2B). Stance phase was significantly reduced (F_(2,77)_ = 14.98; p<0.001; ANOVA) to 90% ±2% (p = 0.012) and 82% ±2% of baseline after 14 and 30 days (p<0.001; Figure 2B). The aggregated parameter duty cycle expresses the stance phase as a percentage of the step cycle (stance + swing phase; Figure 2A). Duty cycle of the hind paws was decreased to 94% ±2% (p = 0.042) after 14 days and 92% ±2% of baseline (F_(2,83)_ = 8,29; p<0.001; ANOVA; Figure 2B) after 30 days. In addition to these dynamic parameters, we observed a reduction of the hind paw print area to 88% ±7% (not significant) after 14 days and 68% ±4% (F_(2,77)_ = 14.98; p = 0.001; ANOVA; Figure 2C) of baseline after 30 days. Other previously reported gait parameters, especially the mean intensity with which the animals place their paws [8], [10], were not significantly different from control animals.

In conclusion, using automated gait analysis we were able to detect changes in the hind paw print area as well as in the duty cycle and stance phases of animals suffering from PNP. No significant changes were observed for forepaw parameters.

### Automated gait analysis shows a moderate correlation with mechanical withdrawal threshold and caudal nerve SNAP

Given the relative simplicity of the Catwalk method, we were interested whether parameters from automated gait analysis would correlate with established measures of sensory PNP such as mechanical withdrawal threshold and caudal nerve SNAP. When the duty cycle of the hind paws was plotted against the corresponding sensory threshold, there was a small degree of positive correlation with a pearsons correlation coefficient (pcc) of 0.47 (r^2^ 0.22; p = 0.002; Figure 2D). Likewise we observed a small negative correlation between swing phase and mechanical threshold (pcc −0.41; r^2^ 0.16; p = 0.008). Correlations with print area and stance phase were in the same order of magnitude, with a pcc of 0.38 (r^2^ 0.14) and 0.47 (r^2^ 0.22) respectively (both p<0.05). In consideration of the strong positive correlation between mechanical threshold and caudal nerve SNAP, we next tested correlations between gait parameters and the caudal nerve SNAP. Again a small positive correlation was observed for the stance phase and duty cycle of the hind paws compared with the caudal nerve SNAP (pcc of 0.42 and 0.38; r^2^ 0.18 respectively 0.14; both p<0.05; Figure 2E).

In summary, the parameters obtained with automated gait analysis correlated only marginally with typical hallmarks of sensory PNP such as mechanical withdrawal threshold and caudal nerve SNAP.

### Effects of gabapentin treatment on mechanical allodynia and gait alterations in paclitaxel induced neuropathy

**Figure 3:**
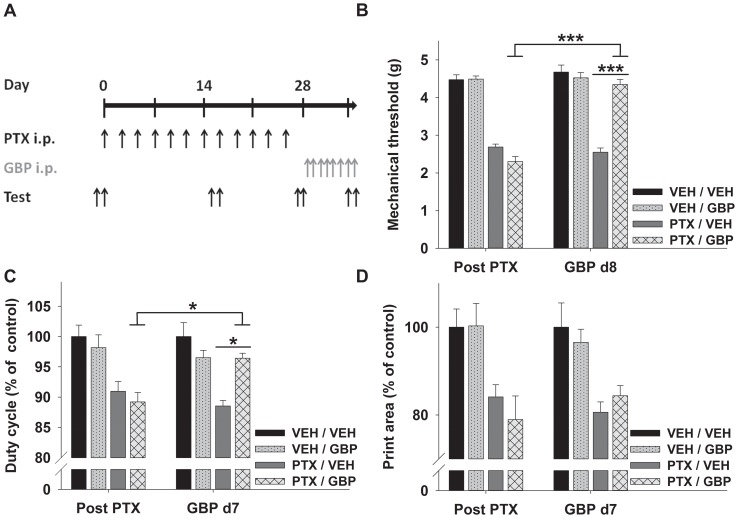
Effects of gabapentin on mechanical allodynia and gait alterations. (A) Schedule of gabapentin (GBP) treatment after the induction of neuropathy with paclitaxel (PTX) and behavioral testing (Test). Animals were allocated to four groups: two groups received paclitaxel and two vehicle (VEH) injections. After the induction of neuropathy, one vehicle and one paclitaxel group were treated with daily gabapentin injections, whereas the other two groups received vehicle. (B) Paclitaxel induced mechanical allodynia was significantly improved after 8 days of gabapentin treatment as well as (C) the dynamic gait parameter duty cycle, whereas (D) the gait parameter hind paw print area was not affected by gabapentin treatment. * p<0.05; *** p<0.001 (Two-way ANOVA).

To further elucidate the relevance of automated gait analysis in the evaluation of paclitaxel induced neuropathy, we tested the effects of “symptomatic” treatment with gabapentin, a ligand of the α2δ1 subunit of voltage-gated calcium channels, on mechanical allodynia and gait parameters. Animals were allocated to four groups and neuropathy was induced as described above in two groups of animals, whereas the other two groups received vehicle injections (Figure 3A). In the paclitaxel treated groups two animals per group had to be sacrificed because of excessive weight loss (>20%). Mice receiving paclitaxel injections moderately lost weight, the difference in mean weight compared to animals receiving vehicle did not exceed 9% ±1% (F_(3,971)_ = 155.6, p<0.001; ANOVA). After the induction of neuropathy, animals underwent behavioral testing as post-paclitaxel baseline. As previous studies reported a more robust treatment effect in rats after several days of gabapentin application [16], an additional round of behavioral testing was conducted after 7 respective 8 days of treatment with 100 mg/kg gabapentin or vehicle per day. Gabapentin treatment almost normalized mechanical allodynia in animals suffering from paclitaxel induced neuropathy (F_(3,105)_ = 104.6; p<0.001; ANOVA). After paclitaxel treatment the mechanical withdrawal threshold, assessed with an electronic von Frey device, was decreased to 56% ±3% (p<0.001) compared to non-neuropathic controls and improved with gabapentin treatment to 93% ±3% (not significant compared to non-neuropathic vehicle control, p<0.001 compared to the post-paclitaxel baseline of the same group, Figure 3B). Neuropathic animals receiving vehicle injections did not show signs of recovery, underlining the effectiveness of gabapentin therapy.

In a next step we evaluated the effects of gabapentin on previously identified relevant dynamic (stance phase, duty cycle) and static (print area) gait parameters. Both dynamic gait parameters improved in neuropathic animals undergoing gabapentin treatment: stance phase increased from 86% ±3% to 97% ±2% and duty cycle from 89% ±2% to 96% ±1% (p<0.05, Figure 3C) compared to vehicle treated non neuropathic animals, however only the aggregated parameter duty cycle reached statistical significance (F_(3,210)_ = 15.14; p<0.001; ANOVA). In contrast to the aforementioned parameters, print area was not influenced by gabapentin treatment (Figure 3D). No spontaneous improvement could be detected in neuropathic animals receiving vehicle injections. Furthermore, gabapentin itself did not induce significant alterations of gait parameters in non-neuropathic animals.

Taken together, gabapentin treatment improved symptoms of sensory neuropathy such as mechanical allodynia and alterations of dynamic gait parameters, whereas no effect on the static gait parameter hind paw print area could be detected.

## Discussion

The aim of this study was to establish a mouse model of dose-dense paclitaxel therapy and to evaluate automated quantitative gait analysis with the Catwalk method as a measure of sensory PNP. Dose-dense paclitaxel therapy with a cumulative dose of 240 mg/kg BW was generally well tolerated: Out of a total of 45 paclitaxel treated animals 4 had to be sacrificed due to excessive weight loss, while mean weight loss compared to vehicle injected mice did not exceed 9%. We observed that the paclitaxel treatment protocol used in this study led to the development of typical features of sensory PNP: the caudal nerve SNAP was markedly reduced and mechanical allodynia with a reduced mechanical withdrawal threshold developed. No significant alteration of the tail nerve conduction velocity was observed. In summary, these findings suggest that animals treated with dose-dense paclitaxel therapy develop a sensory predominantly axonal PNP, which is in line with clinical observations from patients receiving paclitaxel treatment [13] and previous animal models of this condition [2].

Automated gait analysis with the Catwalk method reliably detected 1) a reduction of the hind paw print area as well as a decrease in 2) stance phase and 3) duty cycle of treated animals. Given that paclitaxel induced neuropathy develops in a symmetrical fashion, we analyzed parameters for the left and right side together and for the hind and forepaws separately. Interestingly, no significant changes were observed for forepaw parameters, which is most likely due to the fact that paclitaxel induced PNP is length dependent and most prominent in lower limbs [13]. Taken together, the observed alterations in gait parameters suggest that mice suffering from paclitaxel induced PNP reduce contact of their hind paws with the floor to a minimum. One important question in respect to the observation of gait alterations in animals with paclitaxel induced neuropathy is whether these changes are due to sensory neuropathy or reflect an additional motor neuropathy. Alterations in motor coordination were previously reported in paclitaxel treated animals, although overall motor neuropathy is much less common than sensory neuropathy (reviewed by [29]). In line with these findings, we neither observed clinical signs of paresis nor an impairment of motor coordination in our model. Furthermore, treatment with gabapentin as described previously [16], was able to significantly improve mechanical allodynia and alterations of the parameter duty cycle in neuropathic animals, without inducing alterations of the studied gait parameters in non-neuropathic animals. In summary, the observed gait alterations are most likely due to paclitaxel induced sensory polyneuropathy, as no evidence for a relevant motor neuropathy could be detected.

Our results are similar to previous Catwalk observations in animals with a mononeuropathy following a lesion of the sciatic nerve. These studies observed an increase in the swing phase, a reduced stance phase as well as a reduced print area [8]–[10]. The major difference of the present findings to the aforementioned studies is that the neuropathy in the dose-dense paclitaxel model affects all peripheral nerves to various degrees.

This holds two major methodical challenges: Firstly, it is not possible to normalize data to an unaffected side and thus compare it to an internal control [10]. The lack of an internal control is challenging, because slight alterations of gait parameters were inevitable between different time points despite of extensive training. This is most likely due to environmental factors, as parameters which were not affected by the induction of sensory PNP tended to change concomitantly with each other. In conclusion, automated gait analysis in the PNP model requires the inclusion of a non-neuropathic control group. Secondly, in contrast to unilateral lesions the animals cannot compensate pain in one extremity with their “healthy” side; this likely leads to a smaller effect size. The alterations observed in mice suffering from CIPN were much more subtle than those described in studies of animals suffering from a mononeuropathy, which were in the order of 50% and more [8]–[10]. Apart from the lack of compensation with the contralateral side, the pathology elicited by paclitaxel is also less distinct than a mechanical lesion: It was previously reported that paclitaxel treatment with a cumulative dose of 280 mg/kg BW led to no significant reduction of nerve fiber density [30], whereas chronic constriction injury induces a reduction of myelinated fibers in the sciatic nerve by approximately 70% [31]. In addition, the use of mice instead of rats may also be of relevance, as mice are much lighter than rats and the equipment therefore operates closer to its detection limit. This may also explain why we could not detect significant differences in the mean intensity with which the animals place their paws. Another contributing factor is the limited sensitivity of intensity measurements: Previous studies found that at least 40% weight variation is required for a statistically significant result [11].

In comparison to established parameters like caudal nerve SNAP and mechanical allodynia, alterations of gait parameters developed slower. We observed only a small correlation between gait parameters and the mechanical withdrawal threshold, respectively caudal nerve SNAP. In contrast, caudal nerve SNAP and mechanical withdrawal threshold showed a strong positive correlation. Both the mechanical withdrawal threshold and the caudal nerve SNAP were markedly and significantly reduced after 7 out of 12 paclitaxel injections at the middle time point, whereas alterations in the Catwalk parameters were only significant for duty cycle and stance phase duration. This finding differs from the excellent correlation between mechanical withdrawal threshold and Catwalk parameters which was observed in rats with a chronic constriction injury [8]. Another study of acute inflammatory pain in rats, which induced pain by carrageenan injection into the knee, however detected no significant correlation between mechanical withdrawal threshold and Catwalk parameters [11]. We found that dynamic parameters such as duty cycle or stance phase were more reliable in the detection of paclitaxel induced PNP, than were absolute measurements such as intensity and print area. In our model, treatment of mechanical allodynia with gabapentin improved alterations of duty cycle, however no significant effect could be detected for stance phase and print area. This is in contrast to findings from a study conducted in animals with a unilateral nerve injury like spinal nerve ligation or chronic constriction injury, which only observed an effect of gabapentin on mechanical allodynia, but not gait parameters [32]. Upregulation of the α2δ1 subunit of voltage-gated calcium channels, the pharmacological target of gabapentin, in the spinal dorsal horn and dorsal root ganglia has been shown to play an important role in the development of mechanical allodynia after both mechanical [33]–[36] as well as toxic [16], [37], [38] nerve lesions. It is therefore likely, that gabapentin's mechanism of action is similar in these models. The discrepancy between the study of Piesla and coworkers [32] and our data is thus most likely due to the more pronounced and unilateral nerve damage involving motor fibers in the mechanical lesion models compared with the sensory polyneuropathy induced by paclitaxel. In the light of the aforementioned studies, the results from paclitaxel treated mice underline, that gait analysis with the Catwalk method should be regarded as an additional behavior test rather than as an alternative to the von Frey method. Gait is a complex activity which is highly regulated by motor neurons in the spinal cord, nuclei in the brainstem, the basal ganglia, the cerebellum and the neocortex. It is thus likely that an altered sensory input affects gait in more than one way. The discordant results obtained in different animal models, across different species, however advise caution in the interpretation of the behavioral tests used. The observed improvement of the dynamic gait parameters stance phase and duty cycle, which expresses the duration of stance phase in respect to the step cycle, with gabapentin treatment suggests, that these parameters are related to avoidance of mechanical allodynia in the present model (reviewed by [39]), while others such as print area may reflect a complex response to an altered sensory input.

These results underline the relevance of gait analysis in the assessment of painful sensory PNP; however, while the mechanical withdrawal threshold remains the gold standard for quantifying mechanical allodynia [4], altered gait parameters merit recognition as an additional measure of sensory, but likely also motor, PNP. Although changes in gait patterns correlated only moderately with electrophysiological alterations or mechanical allodynia, they may have clinical implications when new preventive or therapeutic strategies are evaluated.

## Conclusions

We report that dose-dense paclitaxel therapy leads to a painful sensory and axonal PNP in C57BL/6J mice, which is characterized by distinct gait alterations, a reduced caudal nerve SNAP and the development of mechanical allodynia. Treatment of neuropathic animals with gabapentin, a ligand of the α2δ1 subunit of voltage-gated calcium channels, was able to improve mechanical allodynia and alterations of dynamic gait parameters. These findings not only introduce a novel mouse model for studying PNP induced by dose-dense paclitaxel therapy, but also demonstrate the usefulness of automated gait analysis with the Catwalk method in the assessment of sensory PNP. Under the precondition of a well-controlled experimental setup, automated gait analysis serves as an additional objective method to describe behavioral changes due to the development of painful sensory PNP and may therefore be of relevance for future studies.

## Supporting Information

Table S1 **Number of outliers in the catwalk analysis.** n/N specifies the number (n) of outliers in respect to all animals in this group at the specified time point.
(DOCX)
