# Supplementary material for: Assessment of Paclitaxel Induced Sensory Polyneuropathy with “Catwalk” Automated Gait Analysis in Mice
Source: PLoS One. 2013 Oct 15;8(10):e76772. doi: 10.1371/journal.pone.0076772 (PMC3797113; doi:10.1371/journal.pone.0076772)
Supplement: Table S1 — Number of outliers in the catwalk analysis. n/N specifies the number (n) of outliers in respect to all animals in this group at the specified time point. (DOCX) [file pone.0076772.s001.docx]

**Table S1**

|  | **Experiment 1** | | **Experiment 2** | | | |
| --- | --- | --- | --- | --- | --- | --- |
| **Treatment**  **Parameter** | **PTX** | **VEH** | **VEH/VEH** | **VEH/GBP** | **PTX/VEH** | **PTX/GBP** |
| Duty cycle | d0: 1/15  d14: 1/15  d30: 0/15 | d0: 2/10  d14: 0/10  d30: 0/10 | Post-PTX:  0/15  GBP d7:  1/15 | Post-PTX:  1/15  GBP d7:  1/15 | Post-PTX:  0/13  GBP d7:  0/13 | Post-PTX:  1/14  GBP d7:  0/13 |
| Swing phase | d0: 1/15  d14: 1/15  d30: 1/15 | d0: 0/10  d14: 2/10  d30: 2/10 |  |  |  |  |
| Stance phase | d0: 2/15  d14: 2/15  d30: 2/15 | d0: 1/10  d14: 1/10  d30: 1/10 |  |  |  |  |
| Print area | d0: 2/15  d14: 1/15  d30: 2/15 | d0: 1/10  d14: 0/10  d30: 0/10 | Post-PTX:  1/15  GBP d7:  1/15 | Post-PTX:  2/15  GBP d7:  0/15 | Post-PTX:  0/13  GBP d7:  1/13 | Post-PTX:  1/14  GBP d7:  0/13 |
